# Expression of Estrogen-Related Receptors in Localized Provoked Vulvodynia

**DOI:** 10.1089/biores.2019.0049

**Published:** 2020-02-21

**Authors:** Anu Aalto, Riitta Huotari-Orava, Satu Luhtala, Johanna Mäenpää, Synnöve Staff

**Affiliations:** ^1^ Faculty of Medicine and Health Technology, University of Tampere, Tampere, Finland.; ^2^ Department of Obstetrics and Gynecology, Kanta-Häme Central Hospital, Hämeenlinna, Finland.; ^3^ Fimlab Laboratories, Tampere, Finland.; ^4^ Department of Obstetrics and Gynecology, Tampere University Hospital, Tampere, Finland.

**Keywords:** estrogen-related receptor, estrogen receptor, progesterone receptor, vulvodynia, localized provoked vulvodynia, vulvodynia etiology

## Abstract

Eight percent of women suffer from vulvodynia, a chronic pain condition with unknown etiology. Inflammation and dysregulation of estrogen signaling have been suggested to play a role in the pathogenesis of localized provoked vulvodynia (LPV). Therefore, the aim of the study was to analyze protein expression levels of estrogen-related receptors ERRα, ERRß, ERRγ, estrogen receptor (ERα), and progesterone receptor (PRα) and CD3-positive T cells in the vulvar vestibulum obtained from women suffering from LPV in comparison to healthy, unaffected controls. Vulvar vestibulum tissue specimens were obtained from LPV patients (*n* = 12) who had undergone modified posterior vestibulectomy and from 15 healthy controls. Protein expression of ERRα, ERRß, ERRγ, ERα, and PRα and CD3-positive T cells was analyzed by immunohistochemistry (IHC). Expression of ERRß was significantly more pronounced in samples from LPV compared to healthy controls (*p* = 0.006). No significant difference in the expression patterns of ERRα, ERRγ, ERα, PRα, or CD3 cells was detected. To our knowledge, this is the first study reporting ERR expression in normal vestibulum and in vestibulectomy samples from LPV patients. The higher level of ERRß expression detected by IHC may reflect dysregulation of estrogen signaling in LPV.

## Introduction

Approximately 8% of 18–70-year-old women are estimated to suffer from vulvodynia, a chronic pelvic and vulvar pain condition of unknown origin.^1^ Localized provoked vulvodynia (LPV) is considered to be the most common form of sexual pain in women younger than 30 years of age.^2^ The etiology of LPV is multifactorial and remains mostly unknown.

The role of hormone signaling^3–5^ and inflammation^6^ in LPV has previously been addressed in few studies. It has been suggested that estrogen receptor (ER)α is expressed at significantly higher level in LPV samples compared to healthy controls, while no difference in the expression of ERß and progesterone receptor (PR) A or B was detected.^3^ The findings concerning ERα, PR A, and B expression have been contradictory when samples of primary and secondary LPV have been compared.^4,5^

Similarly, studies reporting the amount of T cells in LPV specimens have been inconsistent.^5–8^ However, there is evidence of deregulated inflammation in LPV since proinflammatory mediators such as tumor necrosis factor-a and interleukin1-b have been shown to be elevated in women with LPV.^6^ In addition, greater numbers of B lymphocytes and mature mucosal IgA-plasma cells with a difference in B and T cell arrangement in germinal centers have been detected in vulvar vestibulum of LPV women.^8^

The estrogen-related receptors (ERRs) are a small family of orphan nuclear receptor transcription factors that are not yet associated with a natural ligand and therefore considered as orphan receptors.^9^ The ERRs were originally discovered due to their similarity to the ERs, however, they do not bind to estrogen itself.^9^ ERR family include three isoforms, ERRα, ERRβ, and ERRγ (American nomenclature committee NR3B1, NR3B2, NR3B3, respectively).^10^

Sequence alignment of ER and ERRα is highly similar; 68% in the DNA-binding domain and moderately similar (36%) in the ligand-binding E domain.^11^ The ERRs are essential factors for normal mitochondrial function.^12^ They also affect many cellular processes by regulating cell energy metabolism, immune response through T cell activation and differentiation, and participate in the estrogen signaling pathway.^13^ High levels of ERRα is found in tissues with high metabolic needs, such as intestinal tract, heart, kidneys, and skeletal muscle.^12^ ERRβ and ERRγ are also expressed in heart and kidney and all three isoforms are expressed in the central nervous system.^12^

Expression of ERRs has been previously studied in benign (lichen sclerosus et atrophicus, LSA), precancerous, and malign vulvar epithelium.^14^ However, previous data are available on ERR isoform expression in vulvar vestibulum neither of healthy women (i.e., no dermatological or pain problems) nor of women suffering from LPV.

The hormonal and inflammatory factors may also be interrelated in the pathogenesis of LPV, since estrogen has been shown to modulate immune response by restricting neutrophil accumulation to the site of inflammation, attenuating the release of proinflammatory mediators and regulating ER gene expression in T-, B-, and dendritic immune cells.^15^ In addition, a wide range of rapid estrogenic actions have been shown on different tissues and cell types by modulating the permeability of different ion channel types^16^ and thereby influencing immune response.^17^

Hormone signaling has been shown to act through transient receptor potential (TRP) channels affecting nociceptor excitability and sensitization in many chronic pain syndromes.^18^ Inflammation is also one possible trigger in peripheral sensitization,^17^ which typically occurs in LPV. Inflammatory mediators can cause an increase in the excitability of peripheral nociceptors^17^ in LPV. Therefore, estrogen signaling could theoretically be actionable in LPV by affecting ion channel permeability, which contributes to nociceptor excitability and sensitization.

Therefore, the aim of this study was to analyze the expression of all three ERR isoforms by immunohistochemistry (IHC) from LPV samples in comparison with samples from healthy women. In addition, ER and PR expression was also studied, and the state of inflammation was also addressed by assessing the total amount of T cells by analyzing the staining of CD3-positive antibody.

## Materials and Methods

This study on LPV patients was carried out at the Department of Obstetrics and Gynecology of Tampere University Hospital (TAUH), Tampere, Finland and the Department of Obstetrics and Gynecology in Kanta-Häme Central Hospital (Hämeenlinna, Finland). The study protocol was approved by TAUH Ethics Committee (5APR2016, R16053), and a written informed consent was obtained from all the healthy controls who volunteered in this study. The Finnish National Supervisory Authority for Welfare and Health gave its permission to use the archival vestibulectomy samples in the present study without consulting the patients.

Twelve modified posterior vestibulectomy samples were collected from the hospital archives (all vestibulectomies performed in TAUH between January 2003 and May 2016). All patients operated had been diagnosed with LPV before surgery according to Friedrich's criteria,^19^ that is, severe pain on vestibular touch or attempted vaginal entry and tenderness on localized pressure within the vulvar vestibule. All patients had received conservative treatments in different combinations for their LPV before operation. The macroscopic and morphological findings of the vestibulectomy specimens were confirmed by an experienced pathologist as a part of routine diagnostics in TAUH department of pathology. Patients with vulvar malignancy, ongoing inflammatory, or skin diseases of vulva were excluded from this study.

As healthy controls, we prospectively recruited 15 healthy volunteers aged 18–40 from Kanta-Häme Central Hospital and TAUH. The exclusion criteria were pregnancy, history of vulvar malignancy, any inflammatory, or skin disease of any part of the body and any type of localized or generalized pain syndrome. Healthy controls were admitted to hysteroscopy for benign reasons (generally hypermenorrhea with a polyp or a fibroid) under general anesthesia or as an office procedure. We used local anesthetic agents (1–2 mL of 0.01% lidocain with adrenalin), and 6 mm punch biopsy from vulvar vestibulum at 7 o'clock was taken. Punch biopsies were routinely embedded in paraffin after a maximum of 24 h of fixation in 10% buffered formalin. All the control biopsies were taken at a standardized time point of menstrual cycle (before cycle day 12).

The demographic data on vulvodynia patients were collected from the hospital register (age, parity, menopausal status, different treatments given before vestibulectomy, and medication). For healthy controls, a short questionnaire containing demographic data, current medication, the phase of the menstrual cycle for the study purposes was filled by a physician at the time of the punch biopsy.

All IHC stainings were performed in the Tampere Histology Facility (HF) at Tampere University. For IHC stainings, formalin-fixed and paraffin-embedded biopsies were routinely processed and cut into 4–5 μm thick serial sections, baked and deparaffinized with n-hexane. Before IHC, standard hematoxylin and eosin (H&E) staining was performed. Before immunostaining, antigen retrieval was done by boiling the slides in TE buffer (50 mM Tris-HCl, 1 mM EDTA pH 9) at +121°C for 2 min. Endogenous peroxidase was blocked by incubating the slides with 3% H_2_O_2_ for 5 min. ER- and PR-stainings were performed with mouse monoclonal antibodies, clones 6F11 (ER) and PGR-312 (PR), both diluted at 1:200 (Leica BioSystems Novocastra Laboratories Ltd., Newcastle Upon Tyne, UK). For CD3 staining, we used anti-CD3e rabbit monoclonal antibody detecting both CD4- and CD8-positive T cells (clone BSR10) at a dilution of 1:200 (Nordic BioSite Ab, Täby, Sweden). IHC- for ERR were performed with mouse monoclonal anti-human ERRα (clone H5844), ERRβ (clone H6705), and ERR-γ (clone H6812) antibodies (Perseus Proteomics, Inc., Tokyo, Japan) that were diluted at 1:500, 1:50, and 1:50, respectively. Sample slides were incubated with primary antibodies for 30 min at room temperature. For the detection, Histofine^®^ Simple Stain MAX PO Multi HRP polymer and Histofine DAB-2V kit (both from Nichirei Biosciences, Inc., Tokyo, Japan) were used according to the manufacturer's instructions. Samples were counterstained with Mayer's hematoxylin with addition of 0.5% CuSO_4_ to intensify the DAB reaction. All the IHC stainings were conducted with an Autostainer 480S immunostainer (Lab Vision Corporation, Fremont, CA). Histologically normal skin and colon tissues were used as positive control samples in the IHC-stainings and were provided from the archives of TAUH Pathology department.

All the stainings were evaluated by an experienced dermatopathologist (R.H.O.) and the first author (A.A.). Immunohistological sections were analyzed under a light microscope (Olympus BX51, Model U-MDOB3, Tokyo, Japan) from representative areas. Staining for ER and PR were scored similar to routine breast pathology using a 0–3 scale: 0 = negative; 1 = <10%; 2 = 11–50%; 3 = 51–100% ( × 20 objective).^20^

Stainings of ERR-receptors were graded using a scale of 0/+/++/+++ (+++ = increased staining compared to control, ++ = stained as control, + = decreased staining compared to control, 0 = unstained/negative) ( × 20 objective). CD3-positive T cells were analyzed by counting the mean number of positive cells per field from 2 to 4 high power fields (hpfs) ( × 40 objective). CD3-cells were graded as 1 ≤ 50 cells/hpf, 2 = 50–100 cells/hpf, and 3 ≥ 100 cells/hpf. The scoring of each section was based on a consensus of two investigators and possible disagreements were resolved by a joint review.

Version 24 of IBM SPSS statistics software was used in statistical analyses (IBM SPSS Statistics for Windows, Version 24.0; IBM Corp. 2016. Armonk, NY). A Fisher's exact test and Mann–Whitney *U*-test were used for statistical comparisons when appropriate. A probability value of *p* < 0.05 was considered as statistically significant.

## Results

Patient demographics are shown in Table 1. The groups were similar with regard to menopausal status and the use of combined and progestin only contraceptives. Healthy controls were older than LPV patients (median age of 39 vs. 27 years, respectively, *p* = 0.016). Also, LPV patients were more often nulliparous than healthy controls (83.3% vs. 40%, respectively, *p* = 0.047).

**Table 1.** Patient Characteristics of Localized Provoked Vulvodynia Patients and Healthy Controls

	LPV patients (n = 12)	Healthy controls (n = 15)	p
Age, median (IQR)	27 (23.25–34.75)	39 (34–44)	0.016
Premenopausal, *n* (%)	11 (91.7)	15 (100)	0.44
Nullipara, *n* (%)	10 (83.3)	6 (40.0)	0.047
Combined contraceptives, *n* (%)	1 (8.3)	2 (13.3)	1.00
Progestin only, *n* (%)	2 (16.7)	1 (6.7)	0.57
Symptom duration in months, median (IQR)	20.5 (12–23.5)	n/a	n/a

IQR, interquartile range; LPV, localized provoked vulvodynia; n/a, not applicable.

**FIG. 1.**
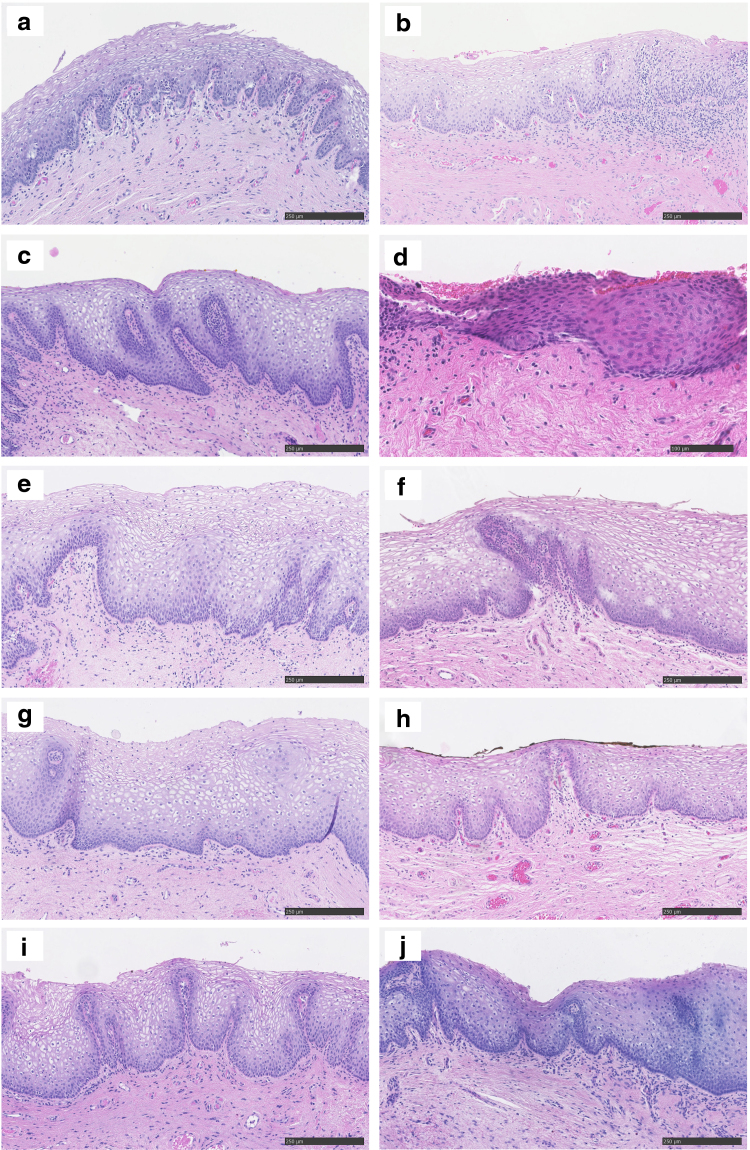

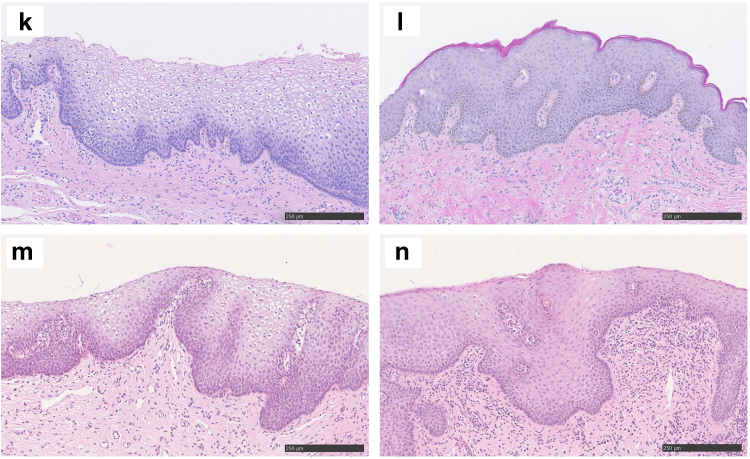
Hematoxylin and eosin stainings of the LPV patients **(a–l)** and examples of two healthy controls **(m, n)**. All the histologic sections were visualized with the 10/20 × objective. LPV, localized provoked vulvodynia.

**FIG. 2.**
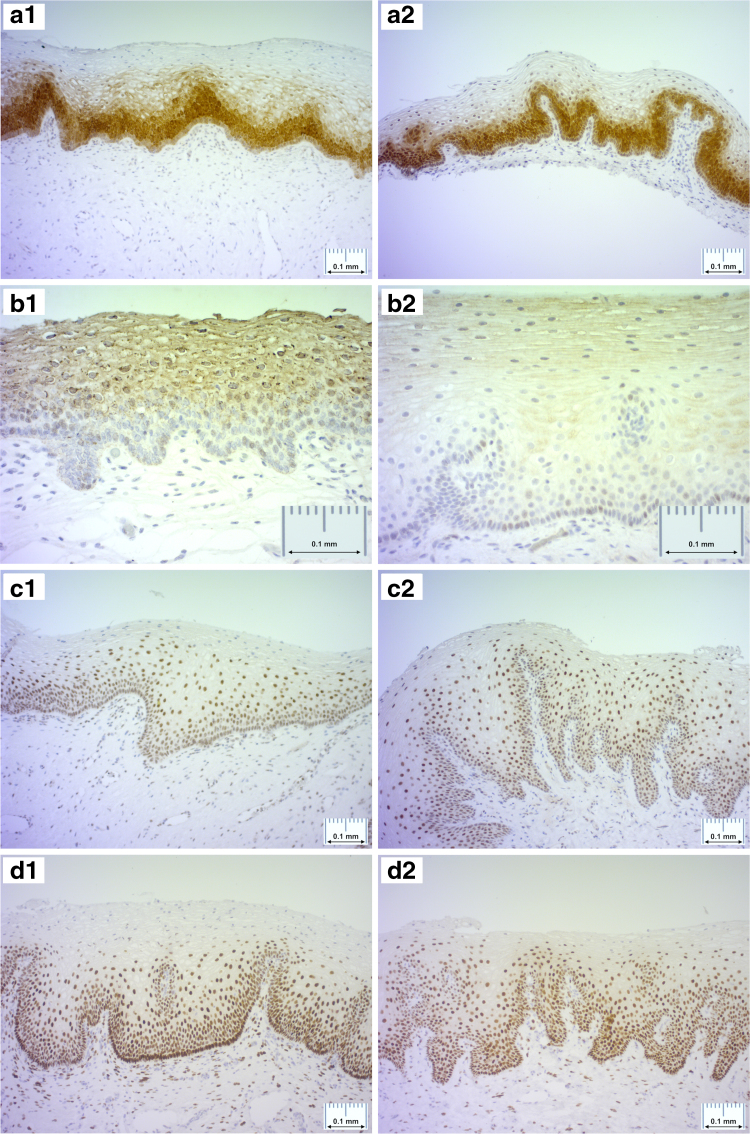

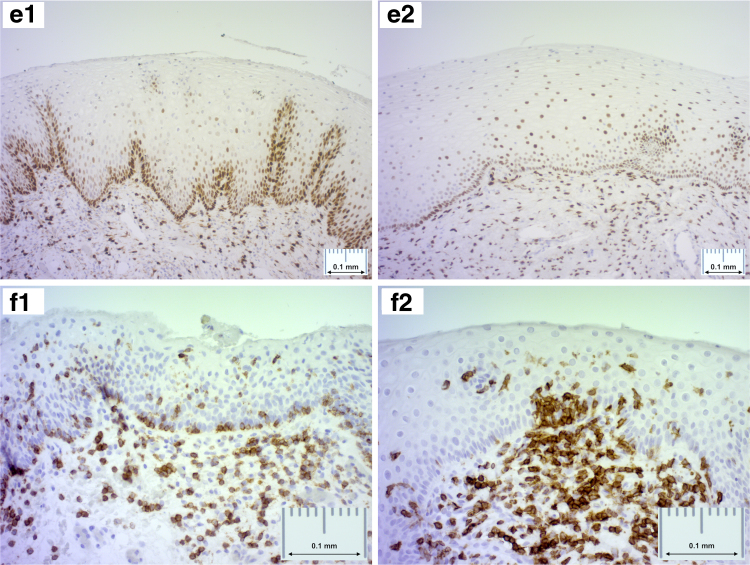
Examples of ERRα immunostaining showing similar cytoplasmic and nuclear staining in vulvar vestibulum from LPV patients **(a1)** and healthy controls **(a2)**. Examples of ERRß immunostaining showing more intense cytoplasmic and nuclear staining in vulvar vestibulum samples from patients with LPV **(b1)** compared to samples from healthy controls **(b2)**. Examples of ERRγ immunostaining demonstrating similar nuclear staining in vulvar vestibulum from LPV patients **(c1)** and healthy controls **(c2)**. Examples of ER immunostaining showing similar expression pattern between samples from LPV patients **(d1)** and healthy controls **(d2)**. Examples of PR immunostaining in vulvar vestibulum samples from LPV patients **(e1)** and healthy controls **(e2)**. Examples of CD3 immunostaining identifying both CD4- and CD8-positive T cells in vulvar vestibulum samples from patients with LPV **(f1)** and healthy controls **(f2)**. All the histologic sections were visualized with the 10/20 × objective using an Olympus BX-51 light microscope (Tokyo, Japan). ER, estrogen receptor; ERR, estrogen-related receptor; PR, progesterone receptor.

The H&E stainings from all the LPV patients in comparison to two healthy controls are shown in Figure 1. No specific pathological diagnostic abnormality was detected in LPV patients or in healthy controls. Chronic nonspecific inflammation was detected in three samples from LPV women (Fig. 1b, f, i). The normal vestibulum from control patients showed uniform staining pattern of all ERR isoforms analyzed. Both in the study and control samples, ERRα and ERRß expression was both nuclear and cytoplasmic, while ERRγ showed only nuclear staining by IHC (Fig. 2). Overall, ERRß staining (both nuclear and cytoplasmic) was statistically significantly more pronounced in LPV samples compared to healthy controls (Fig. 2b1, b2, Table 2, *p* = 0.006). No difference was found in the level ERRα and ERRγ expression (Fig. 2a1, a2, c1, c2, Table 2). Staining of ER, PR, and CD3 was also similar between LPV and control patients (Fig. 2d1–f2, Table 2).

**Table 2.** Stainings of Estrogen Receptor α, Progesterone Receptor α, CD3, ERRα, ERRß, and ERRγ

	LPV patients (n = 12)^a^	Healthy controls (n = 15)^b^	p
ER			
Not stained	0	0	0.181
<10%	0	0	
11–50%	0	2	
51–100%	12	12	
PR			
Not stained	0	0	0.078
<10%	1	5	
11–50%	3	4	
51–100%	8	5	
CD3			
<50 cells/hpf	0	0	0.236
50–100 cells/hpf	1	4	
>100 cells/hpf	10	10	
ERR α			
Stained less than controls	0	0	1.00
Stained as controls	11	14	
Stained more than controls	0	0	
ERR ß			
Stained less than controls	0	0	0.006
Stained as controls	6	14	
Stained more than controls	5	0	
ERR γ			
Stained less than controls	0	0	1.00
Stained as controls	11	14	

^a^ One vestibulectomy sample was sufficient only for ER and PR stainings.

^b^ One of the control samples taken did not contain epithelium, sample was excluded from the analysis.

ER, estrogen receptor; ERR, estrogen-related receptor; hpf, high-power field; PR, progesterone receptor.

## Discussion

We report in this study the expression of ERR isoforms in vulvar vestibulum of LPV patients compared to healthy controls. To our knowledge this is the first study to report the expression of ERRs in relationship to LPV. We also describe the expression of ERRs in the normal vulvar epithelium by IHC. We report here that ERRß expression was more pronounced in the vulva of LPV patients compared to healthy controls. We also report no difference in the expression of ERRα and ERRγ in LPV samples.

We have showed here that normal vulvar vestibulum expressed all ERR isoforms uniformly. Only one previous study has demonstrated the expression of ERR isoforms in normal vulvar skin, but the control/normal population consisted of LSA patients providing control biopsies from normal appearing skin in the vulvar area.^14^ Our findings are consistent with that previous study, but our control samples represent more adequately normal healthy vulvar epithelium since control patients with any history of skin diseases were excluded. There are only few studies addressing ERR expression in normal genital organs such as vagina or endometrium.^21,22^

Previously, Cavallini et al. have described the mRNA expression of ERRs in the premenopausal and postmenopausal human vagina.^21^ They showed a significant decline in ERRα and ERRγ expression in the vaginal epithelium in postmenopausal women, but this was not clearly observed for ERRβ.^21^ ERRβ expression has also been studied in normal human endometrium.^22^ ERRβ mRNA and protein were expressed in healthy human endometrium, but ERRβ protein was mainly localized in the nuclei of both stromal and endometrial cells.^22^ In this study, in contrast, we show that ERRβ was expressed uniformly both in the cytoplasm and nucleus in healthy vulvar epithelium. This may reflect a difference in the functions of ERRβ between normal endometrium and vulva and warrants further investigations.

The expression of ERR isoforms has also been evaluated in certain gynecological disease conditions, but the data reported here regarding expression of ERRs in LPV are novel. ERRα has been shown to decrease in the pathogenesis of vulvar cancer in LSA-positive background.^14^ In contrast, ERRα mRNA was shown to be upregulated in ovarian cancer, while the levels of ERRβ and ERRγ were undetectably low.^23^ The expression of ERRα and ERRγ has also been shown to decrease in endometriotic lesions, but this was not seen in the case of ERRβ.^24^ In endometrial cancer, increased expression of ERRα has been associated with advanced clinical stage and aggressive histology and ERRα silencing resulted in reduced cell proliferation *in vitro.*^25^ All these data imply that dysregulation of ERRs may be active in various gynecological disease conditions.

We have shown here that ERRβ was expressed at higher levels in LPV. The differential expression of ERRβ was not related to differential expression of ER, PR, or to the amount of CD3-positive T cells. In our material, the staining patterns of ER, PR, and the amount of T cells were similar between the study and control samples. Few studies have concentrated on the expression of steroid receptors in LPV. The ER expression in vulvar vestibulum of LPV patients has been previously shown to be both up- and downregulated.^3–5^ The contradictory results obtained may be partly explained by differences in the used study methodology and classification. In this study, we have used the standardized methodology validated in breast cancer diagnostics.^20^ Our finding of similar expression of PR and CD3-positive T cells is consistent with previous studies showing no difference between LPV and control samples.^3,8^

ERRs are known to have an impact on estrogen signaling pathways and immunology, which are both possible etiological factors contributing to the pathogenesis of LPV.^6,15^ Finding that ERRβ is significantly more pronounced in LPV patients' samples may reflect the role of dysregulation of estrogen signaling in LPV. ERRβ has several splice variants and its functions have not been so well understood compared to other ERR isoforms.^26^

Estrogen and sex hormones in general have been shown to play a role in peripheral sensitization by regulating the permeability of different ion channels such as potassium and calcium channels.^17^ Sex hormones have been shown to act through the superfamily of TRP channels, which can act as molecular sensors of chemical and physical stimuli.^18^ Activation of TRP channels in nociceptors result in complex intracellular signaling cascade leading to either neuronal adaptation, that is, desensitization or potentiation.^27,28^ In theory, the present finding of high expression of ERRβ in LPV may suggest that this mechanism can be in actionable also in LPV. However, such conclusion cannot be made from the present data but confirmatory extension studies are needed to resolve the possible role of estrogen signaling in the pathogenesis in LPV.

Limitations of our study include a limited sample size. However, other articles reporting staining patterns of vestibulectomy samples have also been quite limited with respect to sample size.^3,5^ This is mostly due to the relative rarity of the disease and especially the surgical treatment. The study cohort was retrospective generating possible bias with the collection of demographic data. However, the control samples were collected prospectively and it may be regarded as strength of the study. Therefore, the control group consisted only of women who had never suffered from any dermatological or pain problems. In addition, the phase of the menstrual cycle could be standardized in the control group. The study and control groups did not differ with respect to hormonal contraceptive use, which is of importance when studying expression of hormonal factors.

## Conclusions

ERRs are involved in estrogen signaling and in many essential cellular functions. We have shown here that ERRβ expression was increased in vulvar vestibulum from LPV patients. This finding regarding ERRβ expression in LPV warrants further validation in larger, independent LPV cohorts. If validated, ERRβ may serve as a possible target for the future treatments of LPV.

## Abbreviations Used

DAB: 3,3′-Diaminobenzidine
EDTA: ethylenediaminetetraacetic acid
ER: estrogen receptor
ERR: estrogen-related receptor
H&E: hematoxylin and eosin
HF: Histology Facility
hpf: high power field
IHC: immunohistochemistry
IQR: interquartile range
LPV: localized provoked vulvodynia
LSA: lichen sclerosus et atrophicus
PR: progesterone receptor
TAUH: Tampere University Hospital
TRP: transient receptor potential
